# Hematological indices in pediatric patients with acyanotic congenital heart disease: a cross-sectional study of 248 patients

**DOI:** 10.1186/s43042-022-00262-4

**Published:** 2022-03-08

**Authors:** Hanieh Mohammadi, Behzad Mohammadpour Ahranjani, Ehsan Aghaei Moghadam, Farzad Kompani, Mona Mirbeyk, Nima Rezaei

**Affiliations:** 1 Resident Research Committee, Children’s Medical Center, Pediatrics Center of Excellence, Tehran University of Medical Sciences, Tehran, Iran; 2 Department of Pediatric Cardiology, Bahrami Children’s Hospital, Tehran University of Medical Sciences, Tehran, Iran; 3 Pediatric Department, Children Medical Center, Pediatric and Adolescent Cardiovascular Research Center, Tehran University of Medical Sciences, Tehran, Iran; 4 Division of Hematology and Oncology, Children’s Medical Center, Pediatrics Center of Excellence, Tehran University of Medical Sciences, Tehran, Iran; 5 Research Center for Immunodeficiencies, Children’s Medical Center, Tehran University of Medical Sciences, Dr Qarib St, Keshavarz Blvd, 14194 Tehran, Iran; 6 Department of Immunology, School of Medicine, Tehran University of Medical Sciences, Tehran, Iran; 7 Network of Immunity in Infection, Malignancy and Autoimmunity (NIIMA), Universal Scientific Education and Research Network (USERN), Tehran, Iran

**Keywords:** Acyanotic congenital heart disease, Neutropenia, Anemia, Congenital diseases, Pediatrics

## Abstract

**Background:**

Congenital heart disease CHD is a significant cause of mortality and morbidity in children worldwide. Patients with congenital heart disease may develop hematological problems, including thrombocytopenia and neutropenia. In addition, several studies indicate the higher frailty of patients with CHDs to infections and malignancies. Nevertheless, the mechanisms of immune system changes in these patients have remained in the shadow of uncertainty. Moreover, very few studies have worked on cytopenia in CHD. This study has assessed the frequency of thrombocytopenia, neutropenia, lymphopenia, and anemia in pediatric patients with acyanotic congenital heart disease ACHD prior to open-heart surgery.

**Methods:**

This cross-sectional study was handled in the Pediatric Cardiology Clinic, Tehran University of Medical Sciences, during pre-operation visits from 2014 till 2019. Two hundred forty-eight children and adolescents with acyanotic congenital heart disease before open-heart surgery met the criteria to enter the study.

**Results:**

A total of 191 (76.7%) patients with Ventricular Septal Defects (VSD), 37 (14.85%) patients with Atrial Septal Defects (ASD), and 20 (8.11%) patients with Patent Ductus Arteriosus (PDA) were enrolled in this study. The median age was 23.87 months. Thrombocytopenia and neutropenia were found, respectively, in 3 (1.2) and 23 (9.2%) patients. Hemoglobin level and lymphocyte count were significantly lower in patients with neutropenia than patients with normal neutrophil count (*P* value = 0.024 and *P* value = 0.000). Significant positive correlations were found between neutropenia and anemia. There were no correlations between neutrophil count and Platelets. Also, anemia was found in 48 patients (19.3%). The study also found a statistically significant correlation between the co-existence of VSD and neutropenia in the patients (*P* value = 0.000).

**Conclusion:**

Although most were mildly neutropenic, there was a significant correlation between neutropenia and Ventricular Septal Defect compared to PDA and ASD groups. Regarding the importance of neutropenia to affect the prognosis of congenital heart defects in infections, it is important to consider further studies on the status of immune system function in these patients.

## Introduction

Congenital cardiac defects are the most common developmental anomaly, with an incidence of 4 to 10 per 1000 live births. They are the most common noninfectious causes of mortality in pediatrics. CHD unquestionably increases in infants born with Down’s syndrome, as long as newborns of mothers with diabetes mellitus or mothers with congenital heart defects. Regardless of several explained genetic and epigenetic mechanisms that cause congenital cardiac defects, the pathogenesis of most of them has remained unknown [1–5].

Congenital heart defects are comprised of two main groups based on the presence or absence of cyanosis, including acyanotic congenital heart disease [6] and cyanotic congenital heart disease (CCHD) [7]. Ventricular septal defect (VSD) comprises the most significant percentage of cardiac malformations, up to 40% of all congenital heart anomalies [8].

There are studies done to screen children with congenital heart diseases [6] for hematologic abnormalities. Laboratory abnormalities of hematologic tests are common in CCHD patients [9]. Erythrocytosis, thrombocytopenia, platelet function defects, coagulation factors deficiencies are the major hematologic disorders in patients with CCHD [10].

Thrombocytopenia, which usually presents as an incidental finding during a routine evaluation, has been identified as a mechanism of the increased incidence of bleeding, thromboembolism [11], and death in hypoxemic CHD patients [12]. In patients with CCHD, platelets are shown to have both qualitative and quantitative abnormalities [13]. However, there are conflicting data regarding the etiology of thrombocytopenia in CCHD [10].

Although anemia in acquired heart disease is a common problem, little is known in patients with CHD [14]. Anemia is an important risk factor to increase morbidity and mortality in patients with CCHD and ACHD [15]. Heart failure in individuals suffering from ACHD may occur and worsen by anemia as comorbidity [16].

The increased risk of infectious diseases and the immune system dysfunction in children with Down’s syndrome or infants born from mothers with diabetes mellitus who are at a greater risk of CHD can be assumed as a subtle junction between the reduced immune dysfunction and congenital heart defects [17, 18]. Moreover, it is not surprising that there are defined chromosomal disorders disturbing both cardiac structure and immune function, such as 22q11.2 deletion syndrome and Jacobsen syndrome [19–21].

Severe congenital neutropenia (SCN) includes different conditions presenting early in infancy, with recurrent and severe infections. Recently, a novel syndrome associating various organ abnormalities, including cardiac defects, urogenital abnormalities, inner ear hearing loss, and congenital neutropenia, has been described, caused by mutations in the glucose-6-phosphatase catalytic subunit 3 (G6PC3) gene [16].

Furthermore, other than what was mentioned, patients with CHD are more predisposed to cardiac infections and complications of other infections such as Intensive Care Unit hospitalization [22, 23].

This study aimed to assess the prevalence of hematologic disorders in pediatric patients with CHD, compare different hematology parameters between non-hypoxemic CHD patients, and evaluate neutropenia frequency, thrombocytopenia, anemia, leukopenia.

## Materials and methods

### Study design

This cross-sectional study included the hospital records of 248 children and adolescents with CHD < 18 years of age, who had attended the Pediatric Cardiology Clinic, Tehran University during pre-operation visits from January 2014 to December 2019 (128 (51.6%) females and 120 (48.4%) males; aged 1–168 months, mean 23.87). Informed consent was fulfilled and signed consciously by the guardian of each patient before participation. The Ethical Committee approved the procedures applied in this study of Human Experimentation of Tehran University. The ethical code was IR.TUMS.CHMC.REC.1399.191 and was officially notified on February 1st, 2020.

Exclusion criteria were evident sepsis, septic shock, high fever, localized infections, radical operation due to hypoplastic pulmonary arteries, or increased pulmonary vascular resistance due to Eisenmenger syndrome. Patients with congenital cyanotic heart disease and dysmorphic features/DiGeorge syndrome were excluded. No patient was taking or was known to have taken an antiplatelet or anti-inflammatory agent, and none had either Noonan or Down syndrome, in which thrombocytopenia occasionally occurs. None had abnormal liver function tests or hepatosplenomegaly. No platelet transfusion, packed red cells was given prior to hematological analysis. Patients were divided into two groups according to the neutrophil count; ACHD with neutropenia (neutrophil count < 1500 × 10^9^/L) and ACHD with normal neutrophil count (neutrophil count ≥ 1500 × 10^9^/L). This cutoff to diagnose neutropenia is commonly used by physicians in clinics worldwide [24–26]. Patients in neutropenic groups were also divided into three groups according to the severity of neutropenia; mild neutropenia (neutrophil count < 1500 × 10^9^/L), moderate neutropenia (neutrophil count < 1000 × 10^9^/L) and severe neutropenia (neutrophil count < 500 × 10^9^/L). Also, another hematological profile of ACHD patients according to their disease was determined (Table 2). Thrombocytopenia was defined as platelet count < 150 × 10^9^/L. According to patient age, we also assumed anemia as HB less than 2SD [27, 28]. Our investigations from the patients’ hospital records carried out their sex, age at the pre-operation time, echocardiographic data that approved the diagnosis of CHD, and their registered hematological profile one day before cardiac surgery.

### Sample collection and laboratory investigations used in the hospital

Peripheral blood samples were gathered on potassium-ethylene diamine tetra acetic acid (K2-EDTA) (1.2 mg/mL) for complete blood count (CBC). For chemical analysis, clotted samples were obtained, and serum was separated by centrifugation for 15 min at 1000×*g*. CBC was performed using SysmexXt 2000i. A qualitative examination of C-reactive protein with latex agglutination test had been done to see whether the patients had an acute infection or not.

### Echocardiography

All patients underwent an echocardiographic study using a Philips iE33 machine. A standard 2D echocardiogram was done for all patients enrolled in the study using phased array transducers of different frequencies tailored according to each patient’s age, body built, and weight.

### Statistical analysis

The gathered data were analyzed applying Statistical Program for Social Science version 26. Quantitative variables were defined as mean and standard deviation or median and interquartile range (IQR: the difference between 25 and 75th percentiles). Shapiro–Wilk test was employed to determine the normality of data distribution. Qualitative variables were described as number and percent. In order to analyze parametric quantitative variables, a Student t test was performed. The Mann–Whitney test was employed to statistically compare nonparametric quantitative measures between two groups. A *P* value < 0.05 was considered significant in all analyses.

## Results

### Clinical and laboratory characteristics of the ACHD patients

Two hundred forty-eight patients, including 120 (48.4%) male and 128 (51.6%) female that met the criteria to enter the study, were categorized into three main subgroups according to their diagnosis based on an echocardiographic study: 191 (77%) patients with Ventricular septal defect (VSD), 37 (14.9%) patients with Atrial septal defect (ASD), And 20 (8.1%) with Patent Ductus arteriosus (PDA). We categorized the patients according to their principal diagnosis, which means to avoid duplicating the data if the patient had the combination of VSD and any other cardiac anomaly including PDA or VSD, we consider it in the VSD group. Moreover, the patients with ASD + PDA were categorized in the ASD group. The patients’ age ranged from 1 to 168 months with a mean age of 23.9 months. The demographic data and hematologic profile of patients are shown in Table 1.

**Table 1:** Clinical data and Hematological profile of ACHD patients with neutropenia compared with a normal Neutrophil count

Variables	Patients with ACHD neutropenia (*n* = 23)	Patients with ACHD Normal Neutrophil count (*n* = 225)	Total	P value
Age (months), Median (IQR)	7 (11)	11 (22)	10 (19)	0.06
Weight (Kg), Median (IQR)	6 (5.3)	7 (6)	6.8 (6)	0.66
Sex, *n* (%)				0.7
Male	12 (52.2)	108 (48)	120 (48.4)	
Female	11 (47.8)	117 (52)	128 (51.6)	
Type of CHD, *n* (%)				0.015*
VSD	22 (95.6)	169 (75.1)	191 (77)	0.000*
ASD	0 (0)	37 (16.4)	37 (14.9)	0.000*
PDA	1 (4.4)	19 (8.4)	20 (8.1)	0.49
WBC count (× 10^9^/L), Mean (SD)	7.6 (2.1)	10.3 (3.6)	10.1 (3.6)	0.000*
Hemoglobin (g/dL), Mean (SD)	11.1 (1.5)	11.9 (1.5)	11.8 (1.5)	0.005*
Platelet count (× 10^9^/L), Mean (SD)	328 (103)	325 (102)	325.6 (102)	0.89
Anemia, *n* (%)	12 (52.2)	36 (16)	48 (19.3)	0.000*
Leukopenia, *n* (%)	2 (8.1)	1 (0.4)	3 (1.2)	0.184
Thrombocytopenia, *n* (%)	0 (0)	3 (1.3)	3 (1.2)	0.74

None had abnormal liver function tests or hepatosplenomegaly. None of the patients complained of overt bleeding manifestations. Anemia was the most frequent, including 19.3% of patients, followed by neutropenia with 9.3% of all patients; leukopenia and thrombocytopenia were the least frequent.

This study also scrutinized the hematologic profile status, including neutropenia (mild, moderate, and severe), Thrombocytopenia, Anemia, Leukopenia, Lymphopenia, Thrombocytosis in the context of detailed cardiologic diagnosis patients (Table2).

**Table 2:** Hematological disorders of ACHD patients according to their disease

Variables	Frequency	Mild neutropenia	Moderate neutropenia	Severe neutropenia	Thrombocytopenia	Thrombocytosis	Anemia	Leukopenia	Lymphopenia
lVSD, *n* (%)									
VSD	33 (13.3)	1 (0.4)	0 (0)	0 (0)	0 (0)	2 (0.8)	4 (1.6)	0 (0)	0 (0)
VSD-PH	110 (44.2)	12 (4.8)	3 (1.2)	0 (0)	0 (0)	6 (2.4)	23 (9.2)	1 (0.4)	1 (0.4)
VSD-PS-PH	1 (0.4)	0 (0)	0 (0)	0 (0)	0 (0)	0 (0)	0 (0)	0 (0)	0 (0)
VSD-AI	18 (7.2)	3 (1.2)	0 (0)	0 (0)	0 (0)	1 (0.4)	5 (2)	0 (0)	0 (0)
VSD-COA	1 (0.4)	0 (0)	0 (0)	0 (0)	0 (0)	0 (0)	0 (0)	0 (0)	0 (0)
VSD-PDA	5 (2)	1 (0.4)	0 (0)	0 (0)	1 (0.4)	0 (0)	2 (0.8)	0 (0)	1 (0.4)
VSD-TV cleft repair	1 (0.4)	0 (0)	0 (0)	0 (0)	0 (0)	0 (0)	0 (0)	0 (0)	0 (0)
VSD-RVMB	4 (1.6)	1 (0.4)	0 (0)	0 (0)	0 (0)	0 (0)	0 (0)	0 (0)	0 (0)
MULTI VSD	2 (0.8)	0 (0)	0 (0)	0 (0)	0 (0)	0 (0)	1 (0.4)	0 (0)	0 (0)
VSD-ASD-PH	4 (1.6)	0 (0)	0 (0)	0 (0)	0 (0)	0 (0)	0 (0)	0 (0)	0 (0)
LARGE VSD-PFO-PH	1 (0.4)	0 (0)	0 (0)	0 (0)	0 (0)	1 (0.4)	0 (0)	0 (0)	0 (0)
VSD-PH ± MV Cleft	1 (0.4)	0 (0)	0 (0)	0 (0)	0 (0)	0 (0)	0 (0)	0 (0)	0 (0)
VSD-TR	1 (0.4)	0 (0)	0 (0)	0 (0)	0 (0)	0 (0)	0 (0)	0 (0)	0 (0)
VSD-PDA-PH	7 (2.8)	1 (0.4)	0 (0)	0 (0)	0 (0)	0 (0)	1 (0.4)	1 (0.4)	0 (0)
VSD-ASD	1 (0.4)	0 (0)	0 (0)	0 (0)	0 (0)	0 (0)	0 (0)	0 (0)	0 (0)
Total	190 (76.7)	19 (7.6)	3 (1.2)	0 (0)	1 (0.4)	10 (4)	36 (14.5)	2 (0.8)	2 (0.8)
ASD									
ASD	33 (13.3)	0 (0)	0 (0)	0 (0)	0 (0)	1 (0.4)	7 (2.8)	0 (0)	1 (0.4)
ASD-PS	1 (0.4)	0 (0)	0 (0)	0 (0)	0 (0)	0 (0)	0 (0)	0 (0)	0 (0)
ASD-PAPVC	1 (0.4)	0 (0)	0 (0)	0 (0)	0 (0)	0 (0)	1 (0.4)	0 (0)	0 (0)
ASD-PDA	2 (0.8)	0 (0)	0 (0)	0 (0)	0 (0)	1 (0.4)	0 (0)	0 (0)	0 (0)
Total	37 (14.9)	0 (0)	0 (0)	0 (0)	0 (0)	2 (0.8)	8 (3.2)	0 (0)	1 (0.4)
PDA									
PDA	18 (7.2)	0 (0)	0 (0)	1 (0.4)	2 (0.8)	3 (1.2)	2 (0.8)	1 (0.4)	0 (0)
PDA-PH	2 (0.8)	0 (0)	0 (0)	0 (0)	0 (0)	1 (0.4)	1 (0.4)	0 (0)	0 (0)
Total	20 (8)	0 (0)	0 (0)	1 (0.4)	2 (0.8)	4 (1.6)	3 (1.2)	0 (0)	0 (0)
VSD-ASD-PDA	1 (0.4)	0 (0)	0 (0)	0 (0)	0 (0)	0 (0)	1 (0.4)	0 (0)	0 (0)
Total	248 (100)	19 (7.6)	3 (1.2)	1 (0.4)	3 (1.2)	16 (6.4)	48 (19.3)	3 (1.2)	3 (1.2)

There were fourteen groups of VSD patients with co-malformations of the heart besides those with single VSD. The most frequent disease was the combination of VSD and pulmonary hypertension [3], comprised of 110 patients (44.2%), followed by 33 VSD patients (13.3%).

The comparison of the hematologic profile between three VSD, ASD, and PDA groups is shown in Table3.

**Table 3:** Correlations of hematologic profile in three major categories of heart defects

	Frequency	VSD	ASD	PDA	*P* value
Neutropenia, *n* (%)					
Mild Neutropenia	19 (7.6)	19 (7.6)	0 (0)	0 (0)	0.005*
Moderate Neutropenia	3 (1.2)	3 (1.2)	0 (0)	0 (0)	
Severe neutropenia	1 (0.4)	0 (0)	0 (0)	1 (0.4)	
Thrombocytopenia, *n* (%)	3 (1.2)	1 (0.4)	0 (0)	2 (0.8)	0.001*
Thrombocytosis, *n* (%)	16 (6.4)	10 (4)	2 (0.8)	4 (1.6)	0.037*
Anemia, *n* (%)	48 (19.3)	37 (14.9)	8 (3.2)	3 (1.2)	0.83
Leukopenia, *n* (%)	3 (1.2)	2 (0.8)	0 (0)	1 (0.4)	0.23
Lymphopenia, *n* (%)	3 (1.2)	2 (0.8)	1 (0.4)	0 (0)	0.61
Hb (gr/dL), Mean	11.8 (1.5)	11.8 (1.4)	12.3 (1.9)	11.6 (1.8)	0.1
Platelet count (× 10^9^/L), Mean	325.6 (102)	322 (93)	336 (129)	325 (102)	0.8
WBC count (× 10^9^/L), Mean	10.1 (3.6)	9.9 (3.6)	10 (3.4)	11.3 (3.9)	0.21
Lymphocyte count (× 10^9^/L), Mean	5.5 (2.4)	5.5 (2.4)	4.9 (2.2)	6 (2.7)	0.33
Neutrophil count (× 10^9^/L), Mean	3.4 (2.2)	3.2 (2.3)	3.9 (2.1)	3.7 (1.8)	0.018**

*Pearson Chi-Square asymptotic significance

**Kruskal–Wallis test asymptotic significance

### Neutropenia among patients with ACHD

The patients have been divided into two groups regarding neutrophil count; 23 (9.3%) patients with neutropenia and 225 (90.7%) patients with a normal neutrophil count.

There was no significant difference between two groups of neutropenic and non-neutropenic patients regarding age (*P* value = 0.06), gender (*P* value = 0.7), and weight (*P* value = 0.66).

Twenty-two out of 23 neutropenic patients suffered from VSD, which comprised 11.5% of total VSD patients (*P* value = 0.000). In other words, the frequency of neutropenia was reported significantly more in the VSD group than non-VSD groups and significantly less in the ASD group compared to the rest of the patients, while it was not correlated with PDA.

Additionally, the Mann–Whitney *U* test was employed to examine whether the mean ANC differs in VSD patients from patients without VSD. The mean ANC in the VSD group was meaningfully less than that in the non-VSD group (*P* value = 0.005).

Furthermore, other hematologic features that were assessed between two groups of neutropenic and non-neutropenic patients are presented in Table 1.

Although neutropenia frequency was higher in VSD patients than those without VSD, most of them were mild (*P* value = 0.029). This study also cross-tabulated three major heart defect types with neutropenia severity in four categories: normal neutrophil count, mild, moderate, and severe neutropenia. The Pearson’s chi-square asymptotic significance was obtained as equal as 0.005, which means the severity of neutropenia correlates significantly with the type of heart defect. (Table3).

### Hematologic abnormalities among patients with ACHD

In this study, among all patients, 48 (%19.3) patients had Hb < 2SD for their age (mean 11.81 ± 1.6), which was not correlated with the type of the CHD (*P* value = 0.83). The mean amount of Hb was 11.8 ± 1.4, 12.6 ± 1.9, and 11.6 ± 1.8 among patients with VSD, ASD, and PDA, respectively (*P* value = 0.1).

We also evaluated thrombocytosis in patients with ACHD, defined as platelet count ≥ 450 × 10^9^/L. Thrombocytosis was presented in 16 (6.4%) of the screened patients. Thrombocytopenia was only seen in three patients, including one patient with a combination of VSD-PDA and two with PDA, which means all of these three were suffering from PDA. The mean platelet count was not different between the three groups of CHDs (Table 3).

## Discussion

CHD prevalence has increased over the past three decades due to technological advancement and medical care, diagnosis, and management, while about 12 million people with CHD are now living worldwide [29].

CHD led to a considerable burden on patients’ health, society, and economics as long as imposing 589,479 years lived with disability, in 2017 [30].

Over time, with the increase in life expectancy of CHD patients, its mortality has decreased and slightly has been shifting to non-cardiac reasons, such as infection or malignancies [31–33].

A nationwide population study in Taiwan from 2000 to 2014 has shown that more than half of the causes of death in adults with ACHD are non-cardiac, as well as the results of the nationwide study by Yu et al. in Australia [33, 34]. Naidu et al. reported infection as the leading cause of non-cardiac deaths accounting for 10% of CHD mortality from 1991 to 2015 [35]. Diller et al. has shown that in pneumonia patients who need hospitalization, the co-existence of CHD plays a crucial role in increasing the risk of death and adverse events such as demanding mechanical ventilation [36].

Numerous studies have indicated that viral respiratory infections, including influenza and COVID-19, impose a higher risk of mortality, a longer duration of hospitalization, and more complications and economic costs in patients with CHD than the general population [6, 37–39].

The fact that CHD patients are frailer to infections and malignancies gives a cue that the immune system should be studied more in these patients, especially as the world is struggling with the COVID-19 pandemic [40]. Neutrophils and lymphocytes are essential parts of innate and adaptive immune systems. Interestingly, mutations in some genes such as Notch signaling genes or Ras/mitogen-activated protein kinase signaling genes involved in neutrophil functions have led to heart defects in mice [40]. However, unfortunately, the data about how the development of the immune system mutates in CHD are plainly scarce.

In a very recent study by Sarwar et al. assessing the hematological profile of VSD patients, 33.3% of VSD patients aged between 4 and 5 years had lower counts of WBC count; however, the mentioned study did not evaluate the neutrophil count [41]. Reduced T lymphocyte cell and B lymphocyte cell levels have also been reported in the literature in the past decades [42, 43].

In our study, 9.2 percent of patients had neutropenia. Unfortunately, we did not assess a control group, but considering the prevalence of neutropenia in children in Iran, accounting for 3%, it seems that neutropenia is more common in CHD patients [44].

Comparing the CHD group with neutropenia against those without neutropenia, anemia was more common as long as HB amount and WBC count were significantly lower.

Moreover, neutropenia was significantly more common in patients with ventricular defects compared with the non-VSD group. Our findings suggest that these alterations may emanate from a solitary developmental confounding factor. Some factors may regulate ventricular formation, hematological development, and neutrophil production or activation during the embryonic period.

Coagulopathy is another issue that may complicate CHD with an increased risk for both bleeding and thrombotic complications [45, 46]. Moreover, its mechanisms include abnormal blood flow, endothelial dysfunction, reduced coagulation factors, low platelet count or function, fibrinolysis. Furthermore, low platelet count can put CHD patients at higher risk of death [47]. Previous studies demonstrated that platelet count in patients with cyanotic CHD tends to decrease and develop thrombocytopenia [48]. In a study published by Lill et al. in 2006, the frequency of thrombocytopenia in 105 patients with cyanotic CHD was 25% with an average count of 155 ± 12 × 10^9^/L [48]. In another study by Jensen et al., the mean platelet count of 98 CCHD patients was determined 159 ± 60 × 10^9^/L [49].

In this study, the frequency of thrombocytopenia and thrombocytosis was 1.3% and 6.4%, respectively. This contrast of thrombocytopenia frequency may be because this study assessed acyanotic CHD patients, while previous ones had done with CCHD patients. This study may suggest that thrombocytopenia is not the main problem of these patients, while other hematologic anomalies such as anemia and neutropenia should be more focused on.

Patients suffering from cardiac diseases are frequently reported with anemia, which is highly prevalent without vitamin or mineral insufficiency or other definable reasons [50]. However, it was emphasized that more than one-third of the patients with CCHD had iron deficiency anemia [51], which may have been aggravated by concomitant disorders or a combination of factors [15, 52].

Studies were clear that preoperative anemia, especially in heart diseases, gives rise to postoperative cardiac events, complications, and death [53].

Our study noticed that a remarkable number of patients with ACHD had anemia preoperatively, approving the study performed by Amoozgar et al., in which half of ACHD patients were anemic and undiagnosed due to being asymptomatic [54]. Besides, in the recent study by Sarwar et al., the prevalence of anemia with below-normal hemoglobin varied from 30 to 40 percent in different age groups from 0 to 36 months [41]. It is essential to assess and treat preoperative anemia regarding the significant frequency of anemia in these patients and prevent its complications, morbidity, and mortality. Indeed, physicians commonly misdiagnose anemia because it is often asymptomatic or exhibits largely nonspecific symptoms.

In conclusion, ACHD patients, especially patients with VSD, should be considered a group with a potential risk of neutropenia and be evaluated to avoid remaining misdiagnosed as well as to reduce the risk of infection and its consequences. Besides, this study highly suggests further evaluation to determine the frequency and complications of neutropenia and other hematological impairments in ACHD patients, affecting their prognosis. Finally, future studies should examine the cellular and molecular basis of immune system dysfunction in CHD to pave the way for the health care system to provide these patients an improved quality of life.

## Data Availability

The datasets used or analyzed during the current study are available from the corresponding author on reasonable request.

## Abbreviations

ACHD: Acyanotic congenital heart disease
CCHD: Cyanotic congenital heart disease
CHD: Congenital heart disease
VSD: Ventricular septal defect
ASD: Atrial septal defect
PDA: Patent ductus arteriosus
SCN: Severe congenital neutropenia
